# Successful Administration of Recombinant Human Soluble Thrombomodulin **α** (Recomodulin) for Disseminated Intravascular Coagulation during Induction Chemotherapy in an Elderly Patient with Acute Monoblastic Leukemia Involving the t(9;11)(p22;q23) *MLL/AF9* Translocation

**DOI:** 10.1155/2011/273070

**Published:** 2011-08-02

**Authors:** Kazutaka Takagi, Toshiki Tasaki, Takahiro Yamauchi, Hiromichi Iwasaki, Takanori Ueda

**Affiliations:** Department of Hematology and Oncology, Faculty of Medical Sciences, University of Fukui, Matsuoka Shimoaizuki 23, Eiheiji, Fukui 910-1193, Japan

## Abstract

Patients with acute myelogenous leukemia complicate with disseminated intravascular coagulation (DIC), not only at the time of the initially leukemia diagnosis, but also during induction chemotherapy. In Japan, recently, a recombinant human soluble thrombomodulin alpha (Recomodulin) has been introduced as a new type of anti-DIC agent for clinical use in patients with hematological cancer or infectious disease. We describe a 67-year-old female case in which 25,600 units of Recomodulin for 6 days were successfully administered for both initially complicating and therapy-induced DIC without any troubles of bleeding in an acute monoblastic leukemia (AML-M5a) patient with the *MLL gene* translocation. Furthermore, the levels of DIC biomarkers recovered rapidly after the Recomodulin treatment. Our case suggests that DIC control using Recomodulin is one of the crucial support-therapies during remission induction chemotherapy in patients with acute leukemia of which type tends to complicate extramedullary or extranodal infiltration having potential to onset DIC.

## 1. Introduction

Disseminated intravascular coagulation (DIC), an acquired coagulation disorder that is primarily caused by excessive thrombin formation in systemic microvessels, is a frequent complication of hematologic malignancies, solid tumors, severe infectious diseases, gynecological disorders, and posttraumatic syndrome, which usually require intensive care [1, 2]. Some kinds of acute myelogenous leukemia complicate with DIC, not only at the time of the initial diagnosis, but also during induction chemotherapy, which is frequently disturbed by tumor damage-induced blood coagulopathies including tumor lysis syndrome [3]. Leukemia-released tissue factor (TF) induces endothelial cell disruption, leading to enhanced TF activation in vessel walls or the increased expression of the inflammatory cytokines that upregulate TF activity in endothelial cells or monocytes. To completely cure leukemia, the best supportive care for DIC, including both initially complicating DIC that is present at admission and secondary chemotherapy-induced DIC, is required. However, the current methods for controlling complicating DIC during induction chemotherapy for leukemia are limited, especially for patients being treated in bioclean rooms in hematology/oncology wards. 

Conventional therapies including antithrombin III, protease inhibitors, and heparinoids (standard heparin [4, 5], low molecular-weight heparin [6], and danaparoid sodium [7]) have been widely used as anticoagulant agents for leukemia patients complicated with DIC. Recently, recombinant human soluble thrombomodulin *α* (Recomodulin, Asahi Kasei Pharma, Japan) has been introduced as a new type of anti-DIC agent for clinical use in patients with hematological cancer or an infectious disease in Japan prior to the launch in the world [8]. Physiological thrombomodulin (TM) is a 557 amino acid transmembrane glycoprotein on the vascular surfaces of endothelial cell [9], acts as natural anticoagulant product via various anticoagulant mechanisms and forms a complex with thrombin, which converts protein C to its activated form, which in turn inhibits coagulant factor Va and VIIIa. [10] it On the other hand, TM-mediated thrombin inactivation can chemically inhibit the thrombin-induced activation of fibrinogen and coagulant factor V [11].

Recomodulin is composed of the active, extracellular domain of TM [12]. Recomodulin binds to thrombin to prevent blood coagulation, and the thrombin-Recomodulin complex activates protein C to produce activated protein C (APC), which inactivates factors VIIIa and Va in the presence of protein S, thereby further inhibiting thrombin formation. [8] A phase III, randomized double-blind clinical trial of Recomodulin comparing its efficacy and safety with those of low-dose unfractionated heparin in the treatment of DIC associated with hematologic malignancies or infection showed significant DIC improvement (66.1% of Recomodulin group versus 49.9% of heparin group, *n* = 234) and alleviation of bleeding symptoms [8]. 

*MLL (myeloid/lymphoid leukemia or mixed lineage leukemia)* gene-related abnormalities have frequently been found in many different types of leukemia. [9, 13] Among adult *de novo* AML patients, the frequency of 11q23 rearrangement is 4–7% [14]. *MLL gene* rearrangement was heterogeneously found in 31% of adults with acute monoblastic leukemia (AML-M5a) [15, 16]. Among the *MLL* family of 11q23 rearrangements, there are more than 30 partner genes such as *AF4*, *AF9*, *ENL*, *AF10*, and *ELL* [17]. Among them, the most common partners are 4q21 (*AF4*) in t(4;11) and 9p22 (*AF9*) in t(9;11), which are involved in 40% and 27% of translocations, respectively [18, 19]. *MLL* fusion partners are associated with leukemic blast cells of various lineages [20]: *MLL-AF9* mainly results in AML whereas *MLL-AF4* almost exclusively induces B-cell lineage ALL. The clinical prognosis of patients with 11q23 rearrangement depends on the partner chromosome involved [14]. AML-M5a involving rearrangement of the *MLL* gene is considered to be more likely to present with high-risk features including hyperleukocytosis, extramedullary involvement such as gingival or skin infiltration, and complicating DIC at the time of diagnosis [21]. But, it does not have been well known that *MLL* leukemia usually complicates DIC. Here, we report a case in which a novel promising anti-DIC therapeutic agent, Recomodulin, was successfully administered for initially complicating and therapy-induced DIC in a patient with acute monoblastic leukemia (AML-M5a) displaying *MLL gene* translocation.

## 2. Case Report

### 2.1. Patient

A 67-year-old female was admitted to our hematology/oncology division of university hospital on complaints of systemic lymphadenopathy, pharyngitis, and leukoblastosis combined with thrombocytopenia in January 2010. As past medical history, patient was diagnosed with right breast cancer and subsequently underwent surgery in August 2009, although no anticancer chemotherapy or irradiation therapy was performed. The patient did not have any significant family history of malignant disorders.

### 2.2. Physiological Findings

On admission, the patient's body temperature was 36.1°C, her blood pressure was 126/87 mmHg, and her peripheral pulse was 110 bpm. Systemic purpura and petechiae were seen on her trunk and bilateral lower legs. Marked diffuse swelling of gingival crests (gum hypertrophy) was observed, but no bleeding occurred in the oral cavity. Systemic surface lymphadenopathy was noted, including 3 lymph nodes (LN) in the right submandibular lesion, 2 LNs in the left submandibular lesion, 1 LN at right inguinal lesion. These LNs did not show redness or any tenderness. A CT scan disclosed multiple systemic lymphadenopathies involving the mediastinum, the paraaortic lymph nodes of the upper abdomen, and the paracecum.

### 2.3. Laboratory Data

**Figure 1:**
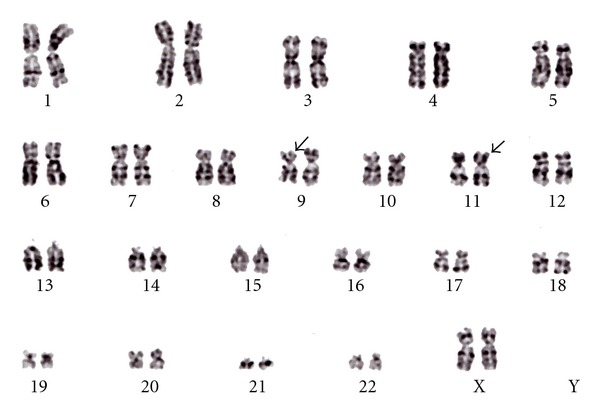
Karyotype analysis showed t(9;11)(p22;q23). At the time of diagnosis, bone marrow cells were analyzed by commercially available service (BML laboratory, Japan). Twenty of the 20 metaphases cells disclosed t(9;11)(p22;q23) in G-banding method.

The patient's peripheral WBC count was 36,900/*μ*L with 74.0% monocytosis, most of which was seen in monoblasts. Her hemoglobin level was 12.2 g/dL, her platelet count was 77,000/*μ*L, and her blood coagulation profile was as follows: PT-INR, 1.45; APTT, 27.0 sec.; FDP, 50.2 *μ*g/mL; D-Dimer, 39.9 *μ*g/mL; TAT, 8.7 ng/mL; PIC, 5.0 *μ*g/mL. From the above data and the patient's bleeding tendency, we diagnosed her as being initially complicated with disseminated intravascular coagulation (DIC). Patient's serum level of CRP or LDH was 3.38 mg/dL or LDH, 1,190 IU/L (normal range, 119–214 IU/L), respectively. Serum and urine lysozyme concentrations increased to 103.0 and 1310.0 *μ*g/mL, respectively, but any other data of blood biochemistry were within normal limits. A bone marrow smear detected 95.8% monoblasts, which were positive for nonspecific-esterase staining in 375,000/*μ*L nucleated cells. The reverse transcriptional-polymerase chain reaction (RT-PCR) was carried out by a commercial laboratory (BML, Kawagoe, Japan) to detect the MLL/AF4 and MLL/AF9 fusion proteins using total RNA extracted from bone marrow cells. The MLL/AF9 fusion protein displayed a copy number of 140,000 copy/*μ*gRNA, but MLL/AF4 was not detected. Chromosome analysis of the patient's bone marrow using the G-band method determined that all of the extracted dividing cells had the t(9;11)(p22;q23) abnormality (Figure 1). From the above data, the patient was diagnosed with acute monoblastic leukemia (M5a in FAB classification) involving *MLL* gene translocation initially complicated with marked DIC prior to chemotherapy.

### 2.4. Clinical Course

**Figure 2:**
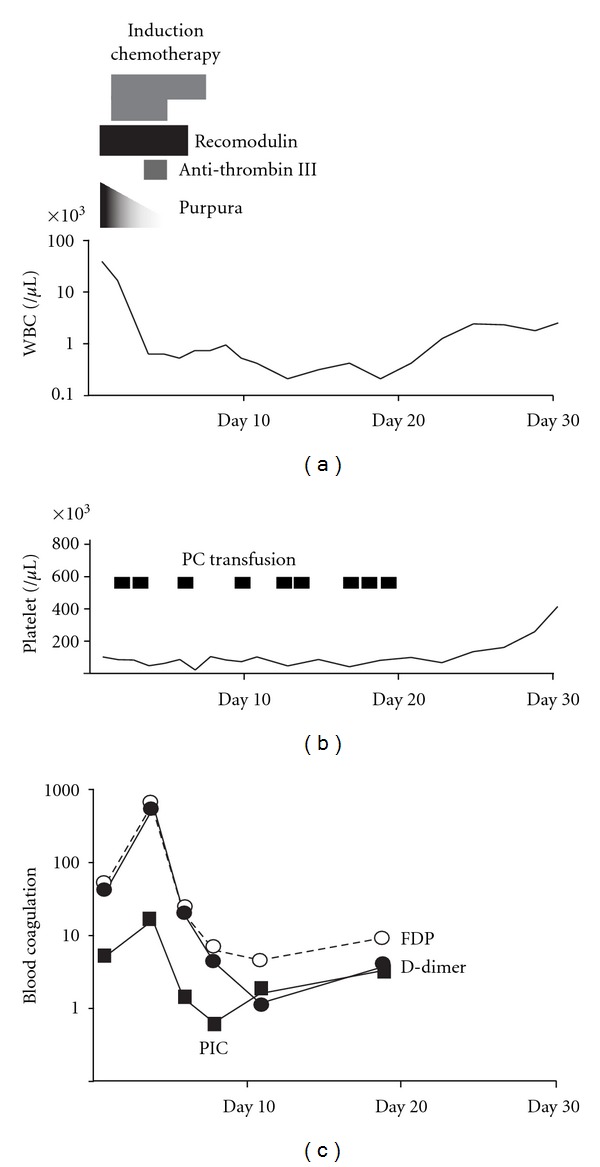
Clinical course. Disseminated intravascular coagulation, DIC, was diagnosed on February 5 in 2010 (the 1st hospital day, day 1), then 25,600 units of recombinant thrombomodulin *α* (Recomodulin) were administered for 6 days. Additional antithrombin III agent was also supplied on days 4 and 5. Several DIC markers rapidly improved on day 6, and bleeding tendency controlled effectively after Recomodulin administration. Adequate times of platelets transfusion (PC transfusion) were needed to overcome DIC or bone marrow suppression after chemotherapy. Patient achieved the 1st complete remission, CR on Mar. 11 in 2010 (day 35). Then, patient has completed 4 courses of consolidation chemotherapy, and the 1st CR has maintained until the present day.

DIC was diagnosed on the first hospital day (Feb 5th 2010; DAY 1), and 25,600 units of recombinant thrombomodulin *α* (Recomodulin) were administered for 6 days (Figure 2). Induction therapy consisting of daunorubicin and araC for acute monoblastic leukemia was started on Feb 6th (DAY 2). Anti-DIC therapy using Recomodulin was effectively administered, not only to control the initial DIC complicating the acute monoblastic leukemia, but also to treat therapy-induced blood coagulation abnormalities caused by the subsequent chemotherapy such as tumor lysis syndrome. The levels of several blood DIC markers measured during the patient's clinical course after the initiation of chemotherapy showed rapid improvement on the 6th hospital day, as follows PT-INR, 1.08; FDP, 5.9 *μ*g/mL; D-Dimer, 4.0 *μ*g/mL; TAT, 5.7 ng/mL; PIC, 0.6 *μ*g/mL. Furthermore, no bleeding tendency was observed after the administration of Recomodulin with adequate PC transfusion support. *Aspergillus* pneumonia occurred during chemotherapy-induced neutropenia and so voriconazole administration was administered effectively. The patient was able to tolerate the induction chemotherapy and achieved her 1st complete remission (CR) on March 11th 2010 (DAY 35). The patient completed 4 courses of consolidation chemotherapy, and the 1st CR has been maintained until the present day.

## 3. Discussion

DIC has been reported to occur in 15–20% of patients with acute leukemia [22], even though it is only seen in 7% of patients with solid cancers [23]. It is considered that leukemia patients receiving remission induction chemotherapy are at high risk of DIC complications [24]. Therefore, controlling DIC is one of the most crucial support therapies administered to leukemia patients during induction chemotherapy. In DIC patients with leukemia, leukemia cells and tumor cells damaged by cytotoxic chemotherapy excrete tissue factor (TF) [3]. The increased TF release initiates the coagulation cascade in the blood [25, 26], and TF serves as a receptor and essential cofactor of factors VII and VIIa, which activate the extrinsic coagulation pathway and require anticoagulation rescue by activated protein C (APC). Thus, cytoplasm-derived TF and insufficient rescue by APC result in DIC in leukemia patients at the time of diagnosis as well as during cytotoxic chemotherapy. Conventional agents including protease inhibitors and heparinoids in combination with the supplementary administration of antithrombin III have been used to support leukemia patients with DIC. However, the worsening of bleeding symptoms in DIC patients, which sometimes occurs during the administration of heparinoid drugs, can be lethal. In Japan, a novel drug recombinant human soluble thrombomodulin alpha (Recomodulin) has recently become available for clinical use in DIC patients with hematological cancer or infectious diseases [8]. Recomodulin forms a complex with thrombin, and the resultant complex converts protein C to the activated form, which inhibits coagulant factors Va and VIIIa of the coagulation pathway [10]. The anticoagulant activity of Recomodulin in DIC patients with leukemia is considered to depend on its APC rescue ability. Taken together, Recomodulin is a novel and promising anti-DIC agent for controlling leukemia- and therapy-associated DIC during remission induction chemotherapy. As another drug for anti-DIC therapy targeting TF-induced coagulopathies, the therapeutic use of recombinant APC has been examined in a clinical trial in DIC patients [27–29].

Mrózek et al. compared adult *de novo* AML patients with the t(9;11) translocation with those with other 11q23 translocations with regard to the type of leukemic blasts that they possessed, their clinical features, and their prognosis [30]. They classified 83% of t(9;11) AML patients into the M5 subtype (FAB) and found that the median CR duration and median survival of t(9;11) AML patients who had been treated with a standard cytarabine and daunorubicin induction regimen were significantly better than those of AML patients with 11q23 translocations (*P* = 0.02 or *P* = 0.009, resp.). They also found that the percentage organ involvement in adult *de novo* AML patients with the t(9;11) translocation was as follows: involvement of the skin, 4%; gum hypertrophy, 21%; splenomegaly, 8%; hepatomegaly, 21%; lymphadenopathy, 17%, but no significant difference was seen compared with 11q23 translocation associated AML [30], suggesting that adult patients with *MLL* leukemia tend to suffer from extramedullary or extranodal lesions; that is, display gingival or skin infiltration, in which cancer infiltration has the potential to activate TF, resulting in DIC [25]. Therefore, strict control of initial DIC or chemotherapy-induced DIC during induction therapy would bring about a better prognosis in patients with 11q23/*MLL *AML. In our case, the blood levels of fibrinolytic markers such as FDP, D-dimer, and PIC increased just after Recomodulin administration, but the levels of these DIC markers subsequently recovered rapidly without any increased bleeding. 

The acquired 11q23 abnormality is associated with therapy-related leukemia and MDS in patients treated with topoisomerase II inhibitors [31]. But, our patient with the 11q23 chromosomal abnormality did not receive any anticancer drugs or suffer from radiation exposure before she was diagnosed with acute leukemia.

## Abbreviation

DIC: Disseminated intravascular coagulation
PT: Prothrombin time
APTT: Activated partial thromboplastin time
FDP: Fibrinogen/fibrin degradation product
TAT: Thrombin-antithrombin complex
PIC: Plasmin-*α*2 plasmin inhibitor complex
AML: Acute myelogneous leukemia
FAB classification: French-American-British classification
TF: Tissue factor
TM: Thrombomodulin
MLL: Myeloid/lymphoid leukemia or mixed lineage leukemia
PC: Platelet concentrate
CR: Complete remission.
